# Supplementary material for: Patterns of Intron Gain and Loss in Fungi
Source: PLoS Biol. 2004 Nov 30;2(12):e422. doi: 10.1371/journal.pbio.0020422 (PMC532390; doi:10.1371/journal.pbio.0020422)
Supplement: Table S1 — Also available at http://genes.mit.edu/NielsenEtAl/. (4.3 MB ZIP). [file pbio.0020422.st001.zip › NielsenEtAl/html/605.html]

AN3714.1.NCU02378.1.MG03529.1.FG07960.1


```
 CLUSTAL W (1.82) Multiple Sequence Alignments - Introns Inserted


Sequence 1: NCU02378.1	607 aa
Sequence 2: MG03529.1	596 aa
Sequence 3: FG07960.1	579 aa
Sequence 4: AN3714.1	594 aa
Alignment Length: 626 aa
Number Identitical Residues: 178 aa
Alignment Score (without introns) 10428


MG03529.1 	MR----RRLQAALVGAVVLGSLPSSILAHGG--ESMNMDHG-QAMVKAA-----QDSDME
NCU02378.1	MKSVTTRPLLAALTGAAVFGLLVPTVLAHGGD-EGGHSGHGGMEMTDAD-----KPRPEE
FG07960.1 	----MIYKKTASRVSNLLFLALLPIVFAHGDSQDMGKMNMGIDHDSNTT-----QSPKHQ
AN3714.1  	-----MLQRISRAALAVFLLASIASALAHGDD-HEMDMNMDMDHMDMGASHENAQPENGD
          	          :  .   .:    .  :***.. .  . . .        : ..::    :

MG03529.1 	----TYFSYPEYQSALYAHIALMIVSWVFMLPVA1VMFSLARSRYTFPAQTVFVVTNLTG
NCU02378.1	EYPLTYFAHPDHKTAIYAHIALMVLAWVFMLPVG1VMLSIARSRYRLLAQFAFLVTNAGG
FG07960.1 	DYPNTYFAHFEHSGVIYAHISLMVISWVFILPIA1VMFSLTRSRFTLPAQLVFLASNTVG
AN3714.1  	DSPMSYFAYGQYTGWIVAHIALMVVAWCFVLPTA1IMLSVARSRMALLSQFIFLVVNAVG
          	. . :**:: ::   : ***:**:::* *:** . :*:*::***  : :*  *:. *  *

MG03529.1 	LILGMIFNENTPDLYPNNAHHKLGWAVTALSCAQVALGVLVRRSEIFGLSLRTRSAR---
NCU02378.1	MLIGIVYNASTPDLYPNNAHHKLGWVVTWLVGAQFAIGLLASVAGVFKKQQSDGNSRERQ
FG07960.1 	VFLGVVYNNNTPDLYPGNAHHKIGWTATWVICAQVLVSVVGYIAGAFRSDESSRETR---
AN3714.1  	LFVGIVYNSQTPDLYENNAHHKIGWVATWVISAQVVMALIFAYAGRGEKVENAYERA---
          	:::*:::* .***** .*****:**..* :  **. :.::   :          .     

MG03529.1 	-YTPVSTAAMAEHQRASAAPGSAQPHRFSFDSGQGTEPNTASLCSRPRSSSFISQHDEEA
NCU02378.1	GFMPVSTQAMEEHN-SHIKPAHNDAYRFSNDSGQGTELSSDAPRSPSLSSSSGAVSRQPL
FG07960.1 	YLLPIPSPEAELPLYSGYHDDS--PCRTSDDTGRGTEPNSPSLQSDSVSTLDGMGSPPQH
AN3714.1  	QFLPVSTDDMAETP-TTLPAGIRHEYRWSRDSGQGTERNTASLHSRASSSCPSPSDEYDG
          	   *:.:        :          * * *:*:*** .: :  * . *:          

MG03529.1 	ARKEEAMLLGGNEFYAHDQEDDLDFGPGRRPS-TRNGAVYRVLALAGKAAGIFSNQVLSI
NCU02378.1	ALQD--VNLHTKEYDDGEDSYDVNSHSGSEPLGPILPASGKAVSIINKIADKISGKFWKV
FG07960.1 	PSKE---------YEDDQDLEELSLSSPAPQG-----------TLARHGSKLATSRVWKH
AN3714.1  	FEKP------DEDIPEQSSESRGFMHTGVLDR-----------FLANRVPSLVSVRALRI
          	  :        ..    ..       .                 :  : .   : :    

MG03529.1 	FALGYNIVDRVILFLGFVAICTGVVTQGRFF0EGQEIFSGLAHWIKGAVFFWLGILTLGR
NCU02378.1	LILAYNVVDRAILPLGFVALCTGIVTYGRFF0EGSGIFSGLAHWIKGGIFFWLGIFTLGR
FG07960.1 	LDIGRKVIDRIILPFGSIVLATGIVTFSRFF0EGDGIFNGLAHWVKGGVFFWLGIFTLGR
AN3714.1  	LNFVYNVIDRLILPLGSIALVSGGVTYGGIM0RDREVFNGLAHFIKGGIFFWYGLITLGR
          	: :  :::** ** :* :.: :* ** . :: ..  :*.****::**.:*** *::****

MG03529.1 	WTGAFAEIGW~AWNLRPAAATAPWRP---SAEFVESALIFIYGSTNIFLEHLGGGADSAW
NCU02378.1	WAGCFGEVGW0AWNVRPKQAGQKWRP---SAEFVESALIFTYGATNVFLEHLG-NWGGAW
FG07960.1 	WSGSFGELGW~AWNVHPKKSLQGWRP---SAELVESFLIFFYGSTNIFMEHLG-GWGGDW
AN3714.1  	MMGCWADLGW~AWNVKPPREIVGWKAKIRSGEFTESAAIFSYGVSNVFLEHLG-GWGGAW
          	  *.:.::** ***::*      *:..  *.*:.**  ** ** :*:*:**** . .. *

MG03529.1 	SAQDLEHVAITVLFIGGGL~CGMLVESATVRELLNTAVADHDEADDHLSTASPLKRQG--
NCU02378.1	SAQDLEHFSITILFIGGGL~CGMLIESVRIRDLLNTTVTEESRQQHHFHDAEVEDSERRE
FG07960.1 	TAQDMEHISITILFLGGGL0CGMLIESTRIRNLLNMTVQDIEPMNPHAVEDANEIKAP--
AN3714.1  	TATDLEHVSIAVLFFGGGL~CGMLFESKRVRNWLNSTVLR-APSRLEAHETANAWNAP--
          	:* *:**.:*::**:**** ****.**  :*: ** :*        .             

MG03529.1 	----SVSLNPIPALVILLLGIMMSSHTQTEMVSGMVHKQWGDLLKGAAVARGLSYVLMYL
NCU02378.1	PETYSFSMNPIPALVILLLGIMMSSHTQDSMISSMVHKQWGNLLTGASFARGFTYVITYL
FG07960.1 	-DTYELSLNPIPALVIMLLGLMMSSHTQHTMMSSMVHKQWGNLLIGASFARGLTYILMFI
AN3714.1  	-DTQEVSLNPMPALIILLLGMMMGGHHQTSMVSSMVHKSWGNMLIGFALARGVTYVLMYL
          	 .: ..*:**:***:*:***:**..* *  *:*.****.**::* * :.***.:*:: ::

MG03529.1 	KPPRSVLPSRPPTELLAAFGLMAGGVIFMAS0SHDTVQGMINQGLDTMFMYTVTMGLVAF
NCU02378.1	KPPRSVYPSRPPTELLTAFGLISGGIIFMAS~AGDTVSGMIHYQLDAMFTYTVTMGLVGL
FG07960.1 	KPPTSVLPSRPPTELLTAFGLISGGIIFMAS0STDTVDGMIHYGLDAMFMYTVTMGLVGL
AN3714.1  	KPPTSYLPSRPPTEIVASFCLISGGLIFMLS0TRNVVEALEYYELDAMFATSIALGLTAF
          	*** *  *******::::* *::**:*** * : :.*..:    **:**  ::::**..:

MG03529.1 	LMAWVVIVMALKGWAVRREARWQVVDRSHIA
NCU02378.1	LMAWVILCIALKGWAVRKEAGRYRN------
FG07960.1 	LMAWIVIVLALKGWAMRIERRCIAG------
AN3714.1  	IMAWEIVLIAIKAWALKRQSRPQLAAYRFPA
          	:*** :: :*:*.**:: :           :
```
